# The War in Europe Viewed From the South: A Global Health Concern

**DOI:** 10.3389/ijph.2022.1605184

**Published:** 2022-07-26

**Authors:** Jimoh Amzat, Oliver Razum

**Affiliations:** ^1^ Department of Sociology, Usmanu Danfodiyo University, Sokoto, Nigeria; ^2^ Department of Sociology, Faculty of Humanities, University of Johannesburg, Johannesburg, South Africa; ^3^ School of Public Health, Bielefeld University, Bielefeld, Germany

**Keywords:** war, Europe, global health, food availability, sanctions, SDGs

The West is entangled in a seeming paradox: it abhors the war in Ukraine but continues to fund it by buying oil and gas from Russia (with Germany as the main Western buyer) and giving billions of dollars in weapon aid to Ukraine (with the United States as the main donor). Although “severe” sanctions have been imposed, they are yet to significantly affect Russia’s ability to continue the war. Presently, Russia’s lower sales volume is compensated by the soaring prices. The Russia-Ukraine war is “global” due to its adverse “cluster and scattered” effects (like the cluster bombs and “scatterable” munitions allegedly used by Russia in Ukraine). The sanctions adversely affect less well-off Westerners through increasing energy and food prices [1, 2] which some governments alleviate through tax reductions and other support measures. Unfortunately, the vulnerable populations of the Global South, with very little leverage to end the war, bear the greater brunt of the sanctions’ adverse effects.

War has been described as a man-made public health problem [3]. Russia’s invasion of Ukraine is a typical example of a catastrophe in the globalized world. It is also an example of manufactured risk; sadly, risk-sharing is unavoidable. The sociologists Ulrich Beck and Anthony Giddens described the world as a risk society [4, 5]. Since the world is socio-spatially compressed, most high-level events will have global consequences. As Beck observed, unequal distribution of risk is a reality as risk often settles at the lower rung of society. Russia’s war in Ukraine is a global event with considerable adverse consequences impacting disproportionately on vulnerable populations, mostly in the Global South.

The primary war victims comprise the deceased, injured and displaced in Ukraine and Russia. Many of the secondary victims or casualties are in the Global South, groaning about the concomitant war-related rise in food and energy costs, weakening the already fragile livelihood system and thus, upsurging the poverty and hunger counts. For instance, the war-related inflation shock will push over one million more Nigerians into poverty [6]. Significant inflation and supply disruption mean that “the world’s poor could be forced to do without (food)” [7]. Worse still, there are limited or no social security benefits to protect the vulnerable populations in the Global South. For instance, only 18% of Africa’s population has adequate access to at least one form of social protection [8]. While there are apparent adverse effects on economic growth and poverty reduction, it is early to estimate the other multiplier effects on education, employment, crime, social unrest, social amenities and well-being, including morbidity, mortality and access to healthcare.

Consequently, as the war lingers and the West imposes sanctions, we all feel the pain—not only or even mainly populations of the West [7]. For the Global South, the war is like a spanner thrown in the wheel of progress toward the Sustainable Development Goals. It is a massive global health concern. War is always a path of inhumanity. Although the greater responsibility rests on Russia to end the invasion, it takes all stakeholders, including NATO and the United States, to stop the war. More days of the war imply more sociopolitical and economic devastations, massively affecting the Global South and disadvantaged population groups everywhere. Stopping this war is a Global Health imperative.
